# Modulation of Redox-Sensitive Cardiac Ion Channels

**DOI:** 10.3390/antiox14070836

**Published:** 2025-07-08

**Authors:** Razan Orfali, Al Hassan Gamal El-Din, Varnika Karthick, Elisanjer Lamis, Vanna Xiao, Alena Ramanishka, Abdullah Alwatban, Osama Alkhamees, Ali Alaseem, Young-Woo Nam, Miao Zhang

**Affiliations:** 1 Department of Pharmacology, College of Medicine, Imam Mohammad Ibn Saud Islamic University (IMSIU), Riyadh 13317, Saudi Arabiaoakhamees@imamu.edu.sa (O.A.); amalaseem@imamu.edu.sa (A.A.); 2 Department of Biomedical and Pharmaceutical Sciences, Chapman University School of Pharmacy, Irvine, CA 92618, USA; agamaleldin@chapman.edu (A.H.G.E.-D.); varnikakb@gmail.com (V.K.); ywnam@chapman.edu (Y.-W.N.); zhang@chapman.edu (M.Z.)

**Keywords:** reactive oxygen species, redox signaling, ion channels, cardiomyocytes, oxidative stress, heart failure, antioxidants, kinase, mitochondria, cardiovascular diseases

## Abstract

Redox regulation is crucial for the cardiac action potential, coordinating the sodium-driven depolarization, calcium-mediated plateau formation, and potassium-dependent repolarization processes required for proper heart function. Under physiological conditions, low-level reactive oxygen species (ROS), generated by mitochondria and membrane oxidases, adjust ion channel function and support excitation–contraction coupling. However, when ROS accumulate, they modify a variety of important channel proteins in cardiomyocytes, which commonly results in reducing potassium currents, enhancing sodium and calcium influx, and enhancing intracellular calcium release. These redox-driven alterations disrupt the cardiac rhythm, promote after-depolarizations, impair contractile force, and accelerate the development of heart diseases. Experimental models demonstrate that oxidizing agents reduce repolarizing currents, whereas reducing systems restore normal channel activity. Similarly, oxidative modifications of calcium-handling proteins amplify sarcoplasmic reticulum release and diastolic calcium leak. Understanding the precise redox-dependent modifications of cardiac ion channels would guide new possibilities for targeted therapies aimed at restoring electrophysiological homeostasis under oxidative stress, potentially alleviating myocardial infarction and cardiovascular dysfunction.

## 1. Introduction

Oxidative stress contributes to the pathobiology of many human diseases, including cardiovascular disorders, neurodegenerative diseases, and metabolic syndromes [1]. Enzymatic and nonenzymatic processes can produce reactive oxygen species (ROS) [2]. ROS are chemically reactive molecules containing oxygen, such as superoxide anions, hydrogen peroxide, and hydroxyl radicals, that arise predominantly as natural byproducts of cellular aerobic metabolism and play key roles in cell signaling and homeostasis [3]. Under normal conditions, ROS are present in small amounts and are essential for regular cellular and mitochondrial functions. Although ROS aid in signaling processes that maintain cellular health and activity, excessive ROS levels often cause severe intracellular damage to cells and, ultimately, entire tissues [2,4,5,6]. Excessive oxidative stress can initiate a cycle of inflammation and further ROS production, thereby worsening disease states [7]. In cardiac tissue, ROS are vital for maintaining cellular homeostasis by regulating cell proliferation, differentiation, and excitation–contraction coupling [8,9]. However, an overproduction of ROS disrupts this balance, leading to oxidative stress that causes cellular and molecular damage, and, ultimately, impaired cardiac function [10].

**Figure 1:**
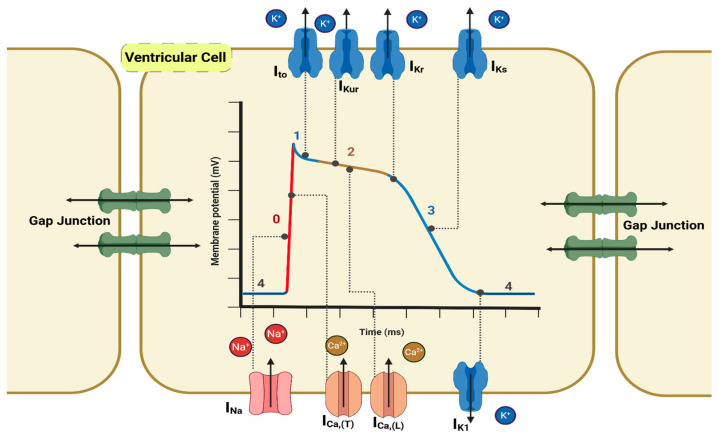
**Simplified ventricular myocyte showing action-potential phases and ion channel localization**. Connexin43-based gap junctions, Igi (green) at intercellular borders, conduct rapid ionic currents between adjacent myocytes, triggering Phase 0 depolarization. This activation opens fast inward currents (red), primarily I_Na_ and I_Ca,T_, which inactivate quickly, while L-type Ca^2+^ channels (I_Ca,L_) remain active longer to sustain the plateau (Phase 2). The depolarizing influence of I_Ca,L_ is then opposed by early outward K^+^ currents (I_to_, blue), and by ultrarapid/delayed rectifier currents (I_Kur_, I_Kr_, I_Ks_), which drive repolarization. Finally, inward rectifier K^+^ current (I_K1_, dark blue) restores the membrane to its resting potential (approximately −85 mV) [25]. Phase numbers (0–4) are marked along the voltage trace.

The heart functions as a biological mechanical pump, ensuring sufficient blood delivery to the body’s organs. Its ability to pump blood depends on the highly coordinated interactions of various cell types [11]. Connective tissue cells provide structural integrity, valve-forming cells construct the heart’s essential atrioventricular and semilunar valves, and cardiomyocytes produce the contractile force necessary for blood circulation [12]. Cardiac function depends on two connected properties: excitability, by which myocytes generate and propagate action potentials, and contractility, where actin–myosin sliding produces force. Tight coupling of these processes underlies the rhythmic pumping, and any disruption can precipitate arrhythmia or pump failure (i.e., heart failure) [13,14,15]. At the cellular level, cardiac excitability arises from a uniquely coordinated process in which ion channels, specialized molecular gates, open and close in a precise sequence, dictating the rhythmic contractions essential for human life (Figure 1) [16,17,18]. Furthermore, the imbalance in Ca^2+^ regulation in the heart significantly contributes to contractile dysfunction and the emergence of irregular heart rhythms within the failing myocardium. Such dysregulation arises from pathological alterations in the expression and activity of a complex group of Ca^2+^-binding proteins, ion channels, and enzymes, which become increasingly elucidated [19,20]. Additionally, excessive production of ROS exacerbates this imbalance, leading to oxidative stress that induces extensive cellular and molecular damage [21]. Most notably, excessive ROS can cause significant changes in the natural double-stranded DNA structure by oxidizing the nitrogenous base guanine into 8-oxoguanine, inducing a cascade of biological mechanisms that can result in apoptosis. Moreover, such cardiac cellular damage ultimately leads to impaired cardiac function [22,23,24]. The redox-sensitive cardiac ion channels respond dynamically to the cellular redox state as critical links between oxidative stress and alterations in cardiac electrophysiology [13]. Understanding the regulation of these channels could offer a clear framework for designing targeted therapies that protect or restore channel function under oxidative challenge.

## 2. Ion Channel Basis of Cardiac Excitation and Conduction

Under physiological conditions, the heart contracts and relaxes at approximately 60 beats per minute. Disruption of this regular rhythm for even a few minutes induces tissue hypoxia, leading to the irreversible injury of vital organs, including the myocardium, and may precipitate sudden cardiac death (SCD) [16,17,26]. The coordinated contraction of the atria and ventricles depends on both rapid action-potential propagation and efficient cell-to-cell coupling (Figure 1) [27,28]. Gap-junctional communication via Connexin43 supports the nearly prompt spread of depolarization, but the redox-sensitive phosphorylation of Connexin43 can slow conduction and promote arrhythmias [29]. This conduction system must remain flexible, capable of rapid heart-rate adjustments, and regulated by autonomic input. Underlying these mechanical events are action potentials (APs), the essential bioelectrical signals that trigger myocardial excitation and contraction [18,30]. The cardiac action potentials are fundamental to myocardial excitation and contraction. Unlike the brief action potentials in neurons and skeletal muscle fibers, cardiac myocytes exhibit a characteristic plateau phase in contractile cells (atria and ventricles) (Figure 1) and spontaneous, rhythmic firing in pacemaker cells (sinoatrial (SA) node [17,18]. These distinctive properties arise from the coordinated activity of voltage-gated ion channels involving sodium (Na^+^), calcium (Ca^2+^), and potassium (K^+^) (Table 1) [16,17].

In contractile myocytes, Phase 0 depolarization is driven by rapid Na^+^ influx through I_Na_, while the plateau phase (Phase 2) results from sustained Ca^2+^ entry through L-type Ca^2+^ channels, facilitating excitation–contraction coupling. Repolarization (Phases 1–3) occurs as Na^+^ and Ca^2+^ channels are inactivated and multiple K^+^ currents, including the delayed rectifier K^+^ currents (I_Kr_, I_Ks_), restore the membrane potential [17,31,32] (Table 1) (Figure 1). The refractory period prevents tetanic contractions and maintains rhythmic pumping. In the SA node, the gradual depolarization during Phase 4 (unstable resting membrane potential) is mediated by the “funny” current (*I_f_*) and transient T-type Ca^2+^ channels, enabling automaticity [18,33,34,35]. Sympathetic stimulation increases the rate of depolarization by enhancing *I_f_* and Ca^2+^ currents, whereas parasympathetic activity slows it through muscarinic receptor-driven K^+^ efflux [18,36]. Impulses generated in the SA node propagate through the conduction system (AV node, Bundle of His, and Purkinje fibers) to synchronize atrial and ventricular contraction. While many studies have linked oxidative stress to cardiac pathology, growing evidence suggests that the redox regulation of ion channels can significantly influence action potential dynamics and may offer novel therapeutic targets [8,22,23,37].

**Table 1:** Main cardiac ion currents and their biophysical roles in action potential phases.

Ion Current	Ion Carried	Principal Subunit (s)	Physiological Role	Reference(s)
I_Na_	Na^+^	SCN5A	Rapid depolarization (Phase 0) and impulse conduction	[38]
I_Ca,L_	Ca^2+^	CACNLIA1	Plateau (Phase 2) and Ca^2+^ entry for excitation–contraction	[39,40,41]
I_to1_	K^+^	Kv_1.2_, Kv_1.4_ Kv_1.5_, Kv_2.1_ Kv_4.2_, Kv_4.3_	Early repolarization (Phase 1), spike-and-dome morphology	[32,42]
I_to2_	K^+^	—	Calcium-activated early repolarization	[32]
I_Kur_	K^+^	Kv_1.5_	Ultrarapid repolarization controls atrial AP duration	[43]
I_Kr_	K^+^	(hERG) Kv_11.1_	Rapid delayed repolarization (Phase 3)	[44]
I_Ks_	K^+^	Kv_7.1_(KvLQT1) + KCNE1	Slow delayed repolarization (Phase 3); repolarization reserve	[45]
I_K1_	K^+^	K_ir2_	Maintains resting potential (Phase 4) and final repolarization	[46]

## 3. Redox Biology in Cardiomyocytes

**Figure 2:**
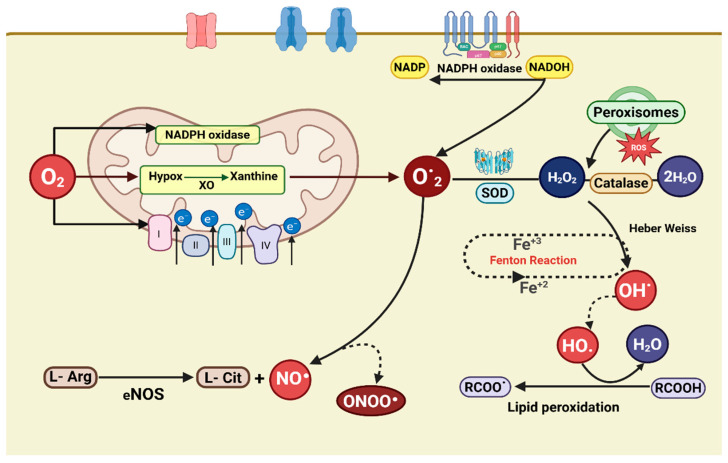
**Primary pathways of reactive oxygen species (ROS) generation in cardiomyocytes**. Within mitochondria, a small portion of electrons “leaks” from complexes I, II, and III onto O_2_, forming the superoxide anion (O_2_·^−^). Superoxide also arises in the cytosol when NADPH oxidase transfers electrons from NADPH to O_2_ and when xanthine oxidase oxidizes hypoxanthine to xanthine. Mitochondrial O_2_·^−^ diffuses into the cytosol, where it can convert hydrogen peroxide (H_2_O_2_) into hydroxyl radicals (·OH) via the Haber–Weiss reaction. Peroxisomes generate much of the cell’s H_2_O_2_, but if peroxisomal defenses fail, H_2_O_2_ spills into the cytosol, fueling further ·OH production. Under metabolic stress, iron released from 4Fe_4S enzyme clusters drives the Fenton reaction, producing additional ·OH from H_2_O_2_. Lipid peroxidation creates membrane peroxyl radicals (ROO·), while excessive nitric oxide (·NO), formed by nitric oxide synthase, combines with O_2_·^−^ to yield the potent oxidant peroxynitrite (ONOO^−^), contributing to nitrosative stress [13].

The heart consumes more oxygen than any other organ. This demand can rise several-fold with increased workload, making it primarily dependent on redox signaling to adjust contractile force acutely and remodel structure constantly [47]. In cardiomyocytes, the primary reactive oxygen species in cardiac cells include the superoxide anion (O_2_·^−^), singlet oxygen (O_2_·), hydrogen peroxide (H_2_O_2_), the hydroxyl radical (OH·), and hypochlorous acid (HOCl) [10,12,13,24]. Key enzymatic sources of oxidative stress in the cardiovascular system are xanthine oxidoreductase (XOR), NADPH oxidases, nitric oxide synthases (NOS), mitochondrial electron-transport chain complexes, and hemoglobin [48,49]. Figure 2 illustrates these pathways: Mitochondrial electron leakage produces most superoxide, while cytosolic enzymes further amplify ROS pools. Adding a single electron to molecular oxygen yields the superoxide radical; because mitochondria use the majority of cellular O_2_ for respiration, most superoxide is produced within the mitochondrial matrix [13]. Mitochondria act as energy generators and signaling centers, using ROS and calcium flux to match contraction to demand. At physiological levels, ROS act as second messengers modulating kinases, transcription factors, and ion channels to adapt cardiac output [4,50,51]. Particularly, Connexin43 hemichannels respond to oxidation: The oxidative modification of critical cysteines disrupts gap-junctional conductance, slowing conduction during ischemia [52,53]. However, the exact redox-sensing mechanisms remain incompletely defined, and some studies report conflicting effects depending on ROS type and cellular setting [54]. When ROS overcome antioxidant defenses, superoxide dismutase (SOD), catalase, and glutathione peroxidase, the resulting oxidative stress triggers lipid peroxidation, protein carbonylation, and DNA damage, provoking inflammation and further ROS generation [55,56]. Critically, most studies rely on high-dose exogenous oxidants, highlighting the need for in vivo models to confirm physiological relevance [57]. Cardiomyocytes ultimately maintain mitochondrial integrity through quality-control mechanisms, such as mitophagy and proteostasis, that can be enhanced by exercise, caloric restriction, or targeted therapies. These interventions reduce oxidative damage and preserve cardiac function [58].

### 3.1. Antioxidant Defense Mechanisms in Cardiomyocytes

**Figure 3:**
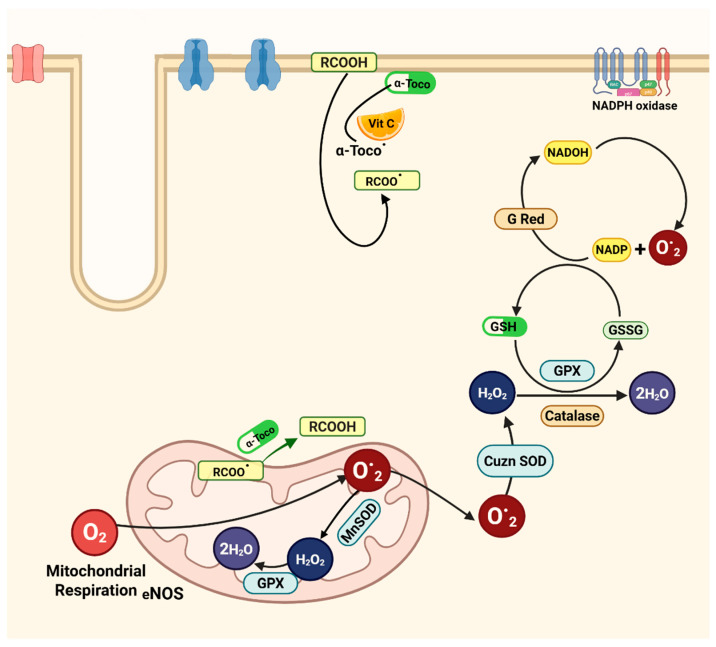
**Antioxidant systems in cardiomyocytes**. Key antioxidants include reduced glutathione (GSH) and its oxidized form (GSSG), supported by antioxidant enzymes such as glutathione reductase (GRed), glutathione peroxidase (GPx), catalase, and superoxide dismutase (SOD). Additional components include NADPH oxidase, α-tocopherol (vitamin E), and vitamin C. Abbreviations: (RCOO^•^) lipid radical; (RCOOH) lipid; (α-toco^•^) α-tocopheroxyl radical; (α-toco) α-tocopherol.

Cardiomyocytes rely on both enzymatic and nonenzymatic systems, such as small-molecule antioxidants, to counteract free-radical damage. Key enzymes include superoxide dismutase (SOD) [59], glutathione peroxidase (GPx) [60], and catalase (CAT) [61]. Small-molecule antioxidants include ascorbic acid (vitamin C), α-tocopherol (vitamin E), glutathione (GSH), carotenoids, and flavonoids [62]. Dietary compounds such as polyunsaturated fatty acids (PUFAs) also modulate cardiac contractility and excitability by enhancing antioxidant capacity [63]. Under resting conditions, a dynamic equilibrium exists between oxidant production and antioxidant activity. For instance, GPx detoxifies peroxides using GSH as a cofactor, converting it to its oxidized form (GSSG) in the process [64]. Figure 3 illustrates how antioxidants work together to maintain redox balance in cardiac cells [13].

### 3.2. Redox Regulation and Cardiac Disease

Redox homeostasis plays a critical role in maintaining cardiac function. In physiological conditions, reactive oxygen species act as signaling molecules that regulate processes such as myocardial contraction [8] and gene expression [65]. This redox signaling is crucial for maintaining cardiac homeostasis. However, the disruption of this balance, characterized by excessive ROS production or impaired antioxidant defenses, leads to oxidative stress, which contributes to the pathogenesis of various cardiac disorders [23,24,66]. Such oxidative stress is associated with conditions like myocardial infarction [67], heart failure [66,68], and atherosclerosis [69].

**Figure 4:**
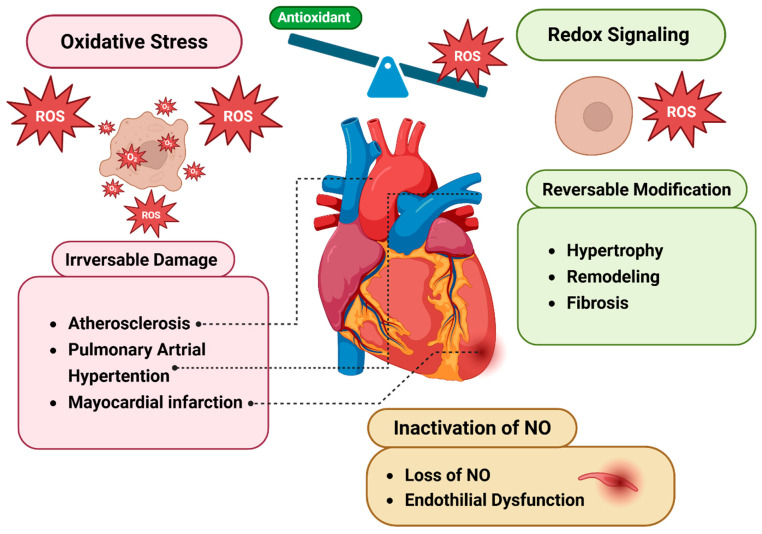
**The impact of oxidative stress on heart diseases.** An imbalance between ROS and antioxidants shifts redox signaling toward oxidative stress, contributing to heart failure, hypertension, and atherosclerosis, often resulting in irreversible cardiac damage. ROS, reactive oxygen species; NO, nitric oxide [73].

Oxidative stress alters ion channel function [21], impairs mitochondrial respiration [4], promotes lipid peroxidation [70], and triggers maladaptive remodeling through the activation of redox-sensitive transcription factors such as NF-κB and AP-1 [71]. These molecular disturbances are linked to the progression of heart failure, ischemia-reperfusion injury, atrial fibrillation, and hypertrophy [24,72]. Emerging evidence suggests that ROS within subcellular compartments, specifically mitochondria, are critical in determining cellular outcomes. Excessive mitochondrial ROS has been observed in cardiomyocytes from experimental models of myocardial infarction and rapid pacing-induced heart failure [73]. ROS can also react with nitric oxide (NO) to produce peroxynitrite (ONOO^−^), which lowers NO availability and disrupts both vascular and cardiac function, a process especially associated with hypertension and heart failure [73,74]. Therefore, understanding the specific redox alterations in cardiomyocytes is essential for identifying therapeutic targets aimed at restoring redox balance and preventing cardiac dysfunction. Figure 4 illustrates how oxidative stress contributes to cardiac damage and disease.

## 4. Redox-Dependent Regulation of Cardiac Ion Channels

Reactive oxygen species influence various signaling cascades in cardiomyocytes [13]. They do so by modifying thiol groups on key regulatory proteins, such as kinases, phosphatases, and transcription factors, that serve as secondary messengers for essential cellular functions [75,76]. Among these, protein kinase C (PKC) is particularly well characterized. In response to ROS, PKC in cardiac cells enhances L-type Ca^2+^ channel opening [77], phosphorylates and activates Na^+^ channels [78,79], and alters various K^+^ currents [80,81,82].

**Figure 5:**
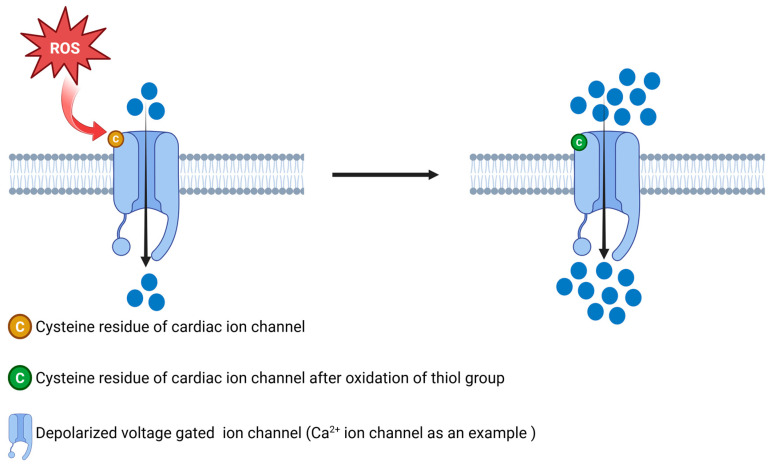
**Redox-dependent modulation of a cardiac ion channel gating.** Reactive oxygen species (ROS) oxidize critical cysteine thiols (orange) on the voltage-sensing domain of the voltage-gated channel, converting them into sulfenic and disulfide forms (green). In the left panel, ROS (red burst) targets the resting channel, initiating thiol oxidation and a partial opening state. These oxidative modifications shift the activation threshold and increase open probability, as depicted on the right by more frequent and longer-lasting ion flux (dense blue dots). Under reducing conditions, thiol groups are restored, and normal gating is recovered.

Intracellular redox balance exerts a profound modulatory effect on ion channel gating. Reactive oxygen and nitrogen species selectively modify pore-forming channel subunits: nitric oxide (•NO) S-nitrosylates critical cysteine thiols [83], peroxynitrite (ONOO^−^) nitrates tyrosine residues [84], and hydrogen peroxide (H_2_O_2_) oxidizes cysteine thiols to sulfenic (–SOH), sulfinic (–SO_2_H), and disulfide (–S–S–) forms [85], as illustrated in Figure 5 [86,87,88]. Functional studies under oxidative conditions have determined distinct biophysical changes in various ion channel families, linking these alterations to cardiac pathophysiological phenotypes (Table 2). This table presents key studies investigating the redox-dependent regulation of cardiac ion channels across diverse experimental models. From rat and mouse cardiomyocytes to heterologous expression systems, these results collectively determine how oxidative modifications, such as glutathionylation, sulfenic acid formation, S-nitrosylation, and disulfide cross-linking, alter the gating expression of potassium, calcium, and sodium channels. Such changes contribute to pathologies ranging from ischemia-reperfusion injury and diabetic cardiomyopathy to arrhythmias and heart failure. In the following section, we will review each redox-sensitive channel in more detail, studying their molecular modification sites, biophysical consequences, and therapeutic implications.

### 4.1. Redox-Sensitive Ion Channels in the Heart:

Redox-sensitive ion channels have emerged as key modulators of the cardiac action potential [103]. These channels respond dynamically to the cellular redox state as critical links between oxidative stress and alterations in cardiac electrophysiology [13]. Redox-sensitive ion channels significantly maintain cardiac rhythm and contractility under normal conditions by influencing cardiomyocytes’ ion flow and electrical signaling. However, under oxidative stress, their altered function can contribute to the development of arrhythmias and further exacerbate cardiac dysfunction [13,20]. ROS effects on various redox-sensitive cardiac ion channels are reviewed below.

#### 4.1.1. Redox Regulation of Cardiac Potassium Channels

Cardiac K^+^ currents are classified by channel topology and distinct functional and pharmacological profiles [104]. Each channel contains four pore-forming α-subunits arranged around a central pore, together with regulatory β-subunits and associated proteins, such as kinases, phosphatases, or cytoskeletal elements, that regulate their gating and cellular localization [16,36]. With various K^+^ channel complexes and varied ROS effects, it is essential to determine ROS sites, whether on channel proteins, auxiliary subunits, or membrane lipids [105]. Acute ROS actions often target specific amino acids (Cys, Met, Tyr, and His), while broader shifts in redox couples (e.g., GSH/GSSG, NADPH/NADP^+^, Fe^2+^/Fe^3+^) can also modulate channel function. Voltage-gated (Kv) channels open upon depolarization and include the transient outward current I_to_, the ultra-rapid I_Kur_, rapid I_Kr_, and slow I_KS_ components of the delayed rectifier, as well as the inward rectifier I_K1_ [30,31]. K_V_ channels are essential for several physiological processes, including muscle contraction, the regulation of resting membrane potential, the control of shape, the duration and frequency of action potentials, and secretion [86,106,107]. Prolonged oxidative stress induces sulfenic acid formation on K_V1.5_, reducing its surface expression, making K_V1.5_ sulfenylation a potential atrial fibrillation target [93]. Other redox-sensitive cardiac potassium channels include K_ATP_ channels, which close in response to intracellular ATP. They assemble from inward rectifier potassium channels (Kir) pore-forming subunits Kir_6.1_ or Kir_6.2_ and regulatory sulfonylurea receptor (SUR) proteins (SUR1, SUR2A, and SUR2B). Because they open when ATP levels fall, K_ATP_ channels sense cellular metabolism and oxygen availability, helping protect the heart during ischemia reperfusion by shortening action potentials, conserving energy, and stabilizing mitochondrial function [91,108]. Calcium-activated K_Ca_ channels are functionally coupled to Ca_v_-mediated Ca^2+^ entry. Small K_Ca_ or SK activation lowers mitochondrial ROS and prevents cysteine oxidation on RyR2 [95]. Leak K^+^ channels conduct background currents independent of voltage or ligand binding. The transient outward current I_to_ is reversibly inhibited by oxidants (e.g., glutathione disulfide, diamide) through disulfide bond formation between key cysteines; reducing agents such as DTT restore I_to_ amplitude [13].

#### 4.1.2. Redox Control of Cardiac Sodium Channels

Voltage-gated Na^+^ channels are also redox targets [97,98,109]. The oxidative modification of extracellular cysteines via disulfide exchange alters toxin and drug binding [86], whereas mitochondrial ROS produced by carbon monoxide donor carbon monoxide-releasing molecule-2 (CORM-2) oxidize intracellular methionine/cysteine residues in Na_V1.5_ to reduce peak current and slow inactivation [98]. Reducing agents such as DTT reverse these effects, whereas small-molecule NO-releasing compounds can modulate the late sodium current via S-nitrosylation [110], suggesting that targeted redox modulation may correct conduction defects in ischemia-related arrhythmias.

#### 4.1.3. Redox Regulation of Cardiac Calcium Channels

Depolarization opens voltage-gated L-type Ca^2+^ channels in the sarcolemma, allowing a small Ca^2+^ influx that activates ryanodine receptors (RyRs) on the sarcoplasmic reticulum in a process called calcium-induced calcium release (CICR) [15]. Cytosolic Ca^2+^ is then cleared by the SERCA pump back into the SR and by the NCX exchanger out of the cell [111]. Reactive oxygen species modulate each of these components, altering overall cellular Ca^2+^ levels [100]. L-type Ca^2+^ channels exhibit complex redox regulation. S-nitrosothiols and SIN-1 enhance Ca_v1.2_ open probability through S-nitrosylation of critical thiols, increasing I_Ca,L,_ and boosting contractility under mild oxidative stimuli [40,100]. In contrast, glutathionylation of Ca_v1.2_, induced by oxidized glutathione in HEK293 expression systems, further potentiates calcium influx during sustained redox shifts, an effect reversible by glutaredoxin mimetics [14,102].

#### 4.1.4. Redox Regulation of Other Ion Channels

Redox regulation significantly affects the function of various ion channels, including chloride channels, which are critical in cardiac physiology [112]. Chloride channels help set the resting membrane potential, shape action potentials, and control cell volume in the heart [113]. Chloride channels are known to respond to redox states, particularly through the modification of cysteine residues within their structure [86]. Mild oxidative environments can induce reversible modifications that enhance the activity of these channels, while excessive oxidative stress can lead to permanent inactivation [114]. Volume-regulated anion channels (VRACs) and Ca^2+^-activated Cl^−^ channels (CaCCs) both sense intracellular redox shifts. VRAC activation under oxidative stress limits ischemia-induced swelling, while redox-dependent modulation of CaCCs influences excitability and arrhythmia risk [115]. Therefore, selective redox-modulating drugs, either thiol-protective antioxidants or targeted channel modulators, would offer promising strategies to correct chloride channel dysfunction in oxidative cardiac disease [116]. By restoring Cl^−^ flux, these mediators could stabilize electrical activity, prevent cell swelling, and improve cardiac outcomes under stress.

## 5. Therapeutic Strategies Targeting Redox-Sensitive Ion Channels in Cardiac Disease

**Figure 6:**
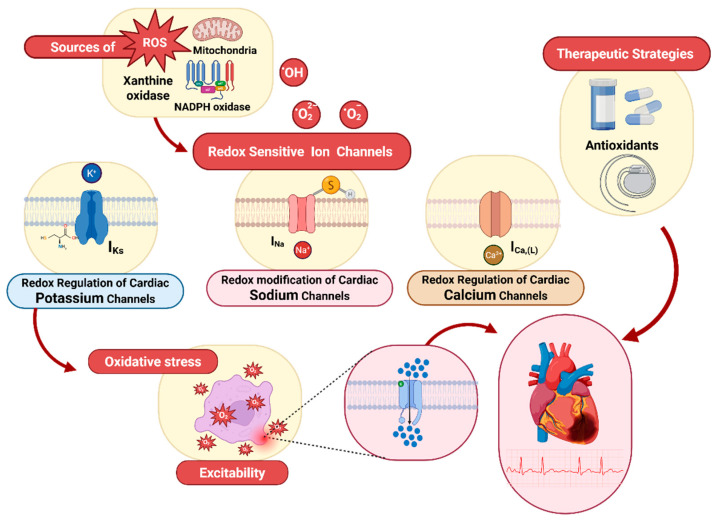
Diagram of redox-dependent modulation of cardiac ion channels and therapeutic interventions. Mitochondrial electron-transport chain leakage, NADPH oxidases, and xanthine oxidase generate reactive oxygen species (ROS: O_2_•^−^, H_2_O_2_, •OH), which impart redox modifications (e.g., S-nitrosylation, glutathionylation, disulfide formation) on voltage-gated Na^+^, Ca^2+^, and K^+^ channels. These thiol-based modifications alter channel gating, leading to disrupted excitability and contractility in ventricular myocytes. Targeted therapeutic strategies, including antioxidants that boost endogenous reductive systems, soluble guanylate cyclase (sGC) stimulators that enhance cGMP-PKG–mediated thiol protection, and device-based pacing (e.g., LBBAP), aim to reverse maladaptive channel oxidation, restore electrical stability, and improve contractile function in oxidative cardiac disease.

Redox-sensitive ion channels represent attractive druggable targets to improve oxidative cardiac injury. Small molecules that stabilize Na_V1.5_ by preventing cysteine oxidation have demonstrated anti-arrhythmic effects under oxidative stress [117]. Similarly, Kv channel modulators ranging from selective peptides to antioxidants such as N-acetylcysteine (NAC) effectively restore repolarization kinetics and suppress redox-driven arrhythmias [118]. NAC additionally substitutes for intracellular glutathione, blocking the disulfide cross-linking of channel thiols and reducing arrhythmia burden in ischemia-reperfusion models [119]. An alternative approach utilizes soluble guanylate cyclase (sGC) stimulators such as vericiguat to increase cGMP signaling, thereby conferring redox protection and vasodilation [120]. Vericiguat was found to inhibit hERG-mediated K^+^ tail currents in a concentration-dependent manner, without evidence of proarrhythmic risk in nonclinical in vitro and in vivo studies [121]. The increase in cGMP also activates PKG, which adds nitric oxide groups (S-nitrosylation) to hKv1.5 channels, reversing harmful thiol oxidation and reducing channel activity [122]. Emerging device-based interventions, notably, left bundle branch area pacing (LBBAP), may synergize with pharmacological redox therapies to reinforce electrical stability in heart failure with reduced ejection fraction (HFrEF) [123,124]. Despite these advances, two critical challenges must be overcome to enable clinical application. First, therapies must achieve cardiac-specific action: systemic delivery of broad-spectrum antioxidants or sGC stimulators risks the off-target modulation of vascular and neuronal ion channels, potentially precipitating hypotension or neurotoxicity [16,125]. Second, the dynamic redox environment of the myocardium and the narrow therapeutic window of ROS/RNS require innovations in targeted delivery and dose optimization [126]. Addressing these issues is key for the successful translation of redox-directed ion channel modulators into standard cardiac clinical practice. Figure 6 provides a conceptual framework that combines a view of how ROS generation, channel-specific redox modifications, and targeted therapies interrelate in oxidative cardiac disease.

## 6. Concluding Remarks

Redox-sensitive ion channels involve a significant network through which oxidative signals shape cardiac excitability and contractile function. Potassium channels respond to thiol-based modifications, adjusting repolarization and protecting against after-depolarizations. Sodium channels combine extracellular and mitochondrial redox signals, modulating conduction velocity and arrhythmia. Calcium channels link redox state to excitation contraction coupling, with balanced S-nitrosylation and glutathionylation preserving contractile strength while preventing diastolic Ca^2+^ leak. Collectively, these findings highlight the contrast of ROS as both physiological mediators and pathological triggers. Reversible redox modifications offer an excellent therapeutic strategy. Compounds that support endogenous reductive systems, selectively reverse harmful channel oxidation, or stimulate soluble guanylate cyclase to bolster cGMP-mediated redox balance hold promise for restoring electrical stability and contractile performance in oxidative cardiac disease. A deep understanding of channel-specific redox mechanisms is essential for the development of precision therapies that restore and sustain electrophysiological homeostasis in oxidative cardiac disease.

## Figures and Tables

**Table 2:** Summary of key studies on redox modulation of cardiac ion channels under oxidative stress. * Indicates clinically relevant, in vivo or translational studies.

Channel Type	Species	Experimental Design	Channel/Subtype	Redox Modification	Related Disease	Effect on Ion Channel	Reference
K^+^ channels	Rats	Patch clamp electrophysiology	Transient outward potassium current (Ito)	Oxidized glutathione (GSSG), 5,5-dithiobis-(2-nitrobenzoic acid)	Ischemia and reperfusion *	Oxidative stress decreases Ito amplitude, reversible by reducing agents	[89]
	Rats	Animal model; spectrophotometric assays; patch clamp electrophysiology	Transient outward potassium current (Ito)	Thioredoxin and glutaredoxin systems	Diabetic cardiomyopathy *	Diabetes alters redox systems, affecting K^+^ channel remodeling	[90]
	Rat ventricular myocytes	Patch clamp; GSSG/H_2_O_2_ vs. GSH/DTT	K_ATP_	GSSG/H_2_O_2_ activates I_KATP_ reversed by GSH/DTT	Ischemia-reperfusion injury	Activation via PKC, PKG, CaMKII	[91]
	Rats	Animal model and in vitro, patch clamp electrophysiology	(Ipeak and Iss)	Diamide, thioredoxin, and glutaredoxin systems	__	Oxidative stress decreases K^+^ currents, regulated by redox systems	[92]
	Mouse ventricular myocytes	In vitro cellular study; patch clamp electrophysiology; Western blotting	Kv_1.5_ (KCNA5)	Sulfenic acid modification	Atrial fibrillation and hypoxic pulmonary hypertension	Sulfenic acid modification of Kv1.5 reduces channel surface expression	[93]
	HEK293 cells	Patch clamp with DTT/GSSG	Kv1.2	Disulfide bond toggling	Arrhythmia	Redox shifts activation voltage	[94]
	Rat	Ex vivo hypertrophic hearts; myocyte patch clamp; mito ROS	SK channels (Small conductance Ca^2^-activated K^+^ channels)	Prevents RyR2 cysteine oxidation	Heart failure/hypertrophy	SK activation lowers mitochondrial ROS and prevents cysteine oxidation on RyR2	[95]
	HEK293 cells	Inside-out patch + H_2_O_2_	BK/Slo1 (Big conductance Ca^2+^activated K^+^ channels)	Cysteine oxidation	Hypertension	ROS inhibit BK via Ca^2+^-sensing cysteines	[96]
Na^+^ channels	Xenopus oocytes	Two-electrode voltage clamp with μO§-conotoxin	Voltage-gated Na^+^ channel (Na_V1.2/1.6_)	Disulfide bond formation at Cys910	Arrhythmia model *	Cys910 redox state controls channel–toxin binding	[97]
	HEK293 cells (human Na_v1.5_)	Heterologous expression; whole-cell patch clamp; CO donor (CORM-2) application; mitochondrial ROS assays	Voltage-gated Na^+^ channel (Na_V1.5_)	Mitochondrial ROS-mediated cysteine oxidation	Ischemia-related arrhythmias *	Carbon monoxide triggers mitochondrial ROS that oxidize Nav1.5 cysteines, reducing peak Na^+^ current	[98]
	Mouse	Ang II infusion; BP telemetry; ROS assay	ENaC (epithelial Na^+^ channel)	NOX1-derived ROS ↑	Hypertension	Noxa1 deletion reduces ENaC activation	[99]
Ca^2+^ channels	Ferret	Patch clamp electrophysiology	L-type calcium channel	SIN-1 (NO and O_2_^−^ donor); S-nitrosothiols	__	NO and S-nitrosothiols modulate L-type Ca^2+^ channel activity	[100]
	Mouse	TRPC6 knockout vs. mice; transverse aortic constriction; ROS assays	TRPC6 (Transient Receptor Potential Canonical 6)	Disrupts TRPC3–Nox2, lowers ROS	Diabetic heart failure	TRPC6 limits ROS, preserves function	[101]
	HEK293 cells	Proteoliposome experiments; patch clamp electrophysiology	L-type calcium channel (Ca_v_1.2)	Thiol-modifying agents (DTT, DTNB)	Ischemia	Oxidative stress modifies Ca_v1.2_ open probability via specific cysteine residues	[102]

## Data Availability

Data sharing is not applicable.

## Abbreviations

AP	Action Potential
Ca^2+^	Calcium Ion
CICR	Calcium-Induced Calcium Release
DTT	Dithiothreitol
ECC	Excitation–Contraction Coupling
GSSG	Glutathione Disulfide
H_2_O_2_	Hydrogen Peroxide
IF	Funny Current (Pacemaker “If” Current)
I_Na_	Fast Sodium Current
I_Ca,L_	L-Type Calcium Current
I_Ca,T_	T-Type Calcium Current
I_K1_	Inward Rectifier Potassium Current
I_Kr_	Rapid Delayed Rectifier Potassium Current
IKs	Slow Delayed Rectifier Potassium Current
IK_ur_	Ultrarapid Delayed Rectifier Potassium Current
I_to_	Transient Outward Potassium Current
ROS	Reactive Oxygen Species
RyR2	Ryanodine Receptor Type 2
SA	Sinoatrial (Node)
SCD	Sudden Cardiac Death
SR	Sarcoplasmic Reticulum
SERCA	Sarco/Endoplasmic Reticulum Ca^2+^-ATPase
TRPC6	Transient Receptor Potential Canonical 6
